# Molecular docking analysis of PPARγ with phytochemicals from Moroccan medicinal plants

**DOI:** 10.6026/97320630019795

**Published:** 2023-07-31

**Authors:** Lamiae Elkhattabi, Salwa Zouhdi, Fairouz Moussetad, Anass Kettani, Abdelhamid Barakat, Rachid Saile

**Affiliations:** Laboratory of Biology and Health, Faculty of Sciences Ben M'Sik, Hassan II University of Casablanca, Morocco; Laboratory of Genomics and Human Genetics, Institut Pasteur du Maroc, Casablanca

**Keywords:** PPARγ, Moroccan phyto-chemicals, virtual screening, molecular dynamic simulation, energy free binding calculation

## Abstract

PPARγ agonists play a crucial role in regulating metabolic homeostasis for treating type-2 diabetes (T2D). Due to the adverse side effects associated with thiazolidinediones, a class of PPARγ agonists, there is a growing interest in identifying natural compounds from medicinal plants that have the potential to bind PPARγ. In this study, we extensively investigated Moroccan phytochemicals using computational structure-based screening with the crystal structure of the PPARγ ligand-binding domain (PDB ID: 7awc) to discover novel phytochemicals targeting PPARγ. The docking results of 540 Moroccan phytochemicals were integrated into online databases for further exploitation through in-depth studies. Drug-likeness analysis was performed to assess the phytochemicals drug-like properties. Two promising phytochemicals, 3,4-dicaffeoylquinic acid and Chlorogenic acid, were identified, both exhibiting high docking affinity and unique binding site interactions compared to the established PPARγ full agonist, rosiglitazone. Molecular dynamics simulations of 100 ns were conducted to examine the stability of the complexes formed by both compounds within the PPARγ active site, and their dynamic behavior was compared to the reference structure of PPARγ alone and with rosiglitazone. Binding free energy calculations demonstrated that 3,4-dicaffeoylquinic acid and Chlorogenic acid exhibited higher binding free energy than the reference agonist, suggesting their potential as candidates for experimental validation in future drug discovery efforts targeting PPARγ for the treatment of T2D and metabolic syndrome.

## Background:

Metabolic syndrome is a cluster of disorders with a high socioeconomic cost that is considered as a worldwide epidemic. Metabolic syndrome is referred to as the simultaneous presence of abdominal obesity, insulin resistance, increased triglycerides (TG), hypertension and reduced high-density lipoprotein (HDL) cholesterol [1- 2,3]. Several definitions for the diagnosis of MetS currently exist [4]. The interconnected physiological, biochemical, clinical and metabolic factors of the MetS directly increase the risk of atherosclerotic cardiovascular disease (ASCVD), T2D and all causes of mortality [5]. Metabolic syndrome will confer a fivefold increase in the risk of T2D and a twofold increase in the risk of developing cardiovascular disease (CVD) in the next five years [6]. Besides this, patients with MetS have a two at fourfold higher risk of stroke, a three at fourfold higher risk of myocardial infarction (MI) and a twofold higher risk of dying from such an event compared to those without syndrome [2] independently of a prior history of cardiovascular events [4]. Moreover, the presence of both MetS and obesity causes chronic low-grade local tissue inflammation and increases susceptibility to other disease conditions such as fatty liver, asthma, sleep disturbances, cholesterol gallstones and some types of cancer [7- 8]. The management of MetS lies in lifestyle modifications to restore energy balance in addition to pharmaceutical interventions. Regarding the treatment of patients with the MetS, employed drugs target different relevant aspects of the MetS such as body weight and fat distribution, insulin resistance, hypertension, dyslipidemia, hyperglycemia or the established prothrombotic and pro-inflammatory state [9]. The peroxisome proliferator-activated receptor γ (PPARγ) is one of the ligand-activated transcription factors in the nuclear hormone receptor superfamily and a pivotal regulator of glucose and lipid homeostasis. PPARγ is an essential regulator of insulin sensitivity, lipid homeostasis, and inflammation and glucose metabolism and therefore it represents an important pharmacological target for drug discovery which can modulate at once several various underlying pathologies of the MetS [10-11]. The PPARγ activators have proven potent in combating hypertension and MetS [12- 13,14]. The thiazolidinediones (TZD) are PPARγ agonists and approved as the first new class of drugs to reduce insulin resistance in patients with T2D [12- 15,16]. The TZD class acts as PPARγ full agonists via the activation function 2 (AF 2) mediated lock mechanism. Troglitazone was the first drug of this class with the potential ability to increase insulin sensitivity and glucose tolerance in obese subjects [17]. This drug was approved in 1997 in the US market as a drug counteracting T2D. It was available until the Food and Drug Administration (FDA) announced its association with the risk of hepatotoxicity and decided to withdraw it in 2000 [18] despite its potential benefits in insulin sensitivity and also in the inhibition of the progression of atherosclerotic lesions, blood pressure reduction as well as decreasing other cardiovascular risk factors [19]. The other two drugs in the TZD class are Rosiglitazone and Pioglitazone which are both used clinically in many countries for glycemic control in the treatment of T2D [20-21]. However, Rosiglitazone was removed from the European market. In the US, the use of Rosiglitazone was restricted by the FDA due to some scientific uncertainty about the cardiovascular safety of its effect. However, the use of Rosiglitazone was associated with a significant increase in the risk of myocardial infarction, as well as a high risk of mortality with cardiovascular diseases [22]. This study delves into drug discovery through the use of computational methods, such as virtual screening, structure-based, molecular dynamics simulations, and free energy calculations. It aims to investigate the interactions between PPARγ protein and Moroccan phytochemicals, with a specific objective to identify natural compounds that exhibit high stability and binding affinity for PPARγ. The researchers then compare their binding residues in their docking pose to that of rosiglitazone in the literature. This research has significant implications for the development of natural alternative activators of PPARγ, as it analyzes the binding residues and functional groups of the identified compounds, providing insight into the underlying mechanisms of PPARγ protein-ligand interactions. Moreover, the focus on Moroccan phytochemicals is particularly noteworthy, as natural compounds represent a valuable and underexplored resource for drug discovery. The identification of potent natural compounds with high binding affinity and stability for PPARγ could lead to potential new therapies for diseases such as type-2 diabetes and metabolic syndrome. Overall, this study provides valuable information for drug discovery and highlights the importance of exploring natural compounds in the search for new treatments.

## Methods:

## Protein preparation:

Crystal structure of PPARγ ligand binding domain in complex with Crystal structure of Peroxisome proliferator-activated receptor gamma (PPARγ) in complex with rosiglitazone (PDB ID 7awc) with a resolution of 1.74 Å was downloaded from Protein Data Bank (PDB). The retrieved structure was prepared for docking through Pymol and PyRx software [23].The protein preparation step entailed the addition of missing residues and atoms and the removal of unwanted metals and water molecules. Finally, hydrogen atoms were added.

## Library of Moroccan phytochemicals:

An intensive literature search was performed to collect a total of 600 phytochemicals originally of various aromatic and medicinal Moroccan plants (Table 1 - see PDF). This collection aimed to explore Moroccan phytocompounds but was not based on any literature criteria related to the diabetes or any related disease, all collected phytochemicals with their plant names, references and useful chemicals details including smiles, molecular formula and 2D structures were included in new moroccan platform naimed MPDB https:/www.mpdb.org for Moroccan phytochemicals database whose is being published soon. 3D structures of each phytochemical were downloaded from PubChem and the pdbqt file corresponding was generated by the Openbabel program.

## Virtual screening:

We performed Docking with the PyRx virtual screening open source program, the binding site of the receptor was defined based on the binding mode of the full agonist rosiglitazone (PDB ID 7awc), During the docking process, the protein was kept rigid and the ligands were flexible with all their torsional bonds free to rotate. A cubic grid box of dimensions 21 Åx29Åx 24Å with points separated by 0.375Å was generated and encompassed all the active site residues of PPARγ.

## Integration of energy binding in online database:

The binding energy values corresponding to the pose with an RMSD of 0Å were determined and subsequently incorporated into a publicly accessible online database, MPDB, available at www.mpdb.org. By disseminating this information, we aspire to galvanize researchers and industry specialists to delve into and exploit the distinctive properties of Moroccan phytochemicals. A manuscript delineating the employed methodology, obtained results, and derived implications has been submitted for review and currently awaits publication.

## Analysis of ligand-receptor interaction:

We used Discovery Studio Visualizer from BIOVIA to analyze the ligand-receptor interactions. This software generates 2D visualization of hydrogen bonding and hydrophobic interactions, which contribute to the affinity of compounds within the active site of PPARγ [24].

## Drug-like properties of the identified hits:

To assess the drug-likeness of the three compounds of interest, we evaluated their physicochemical properties according to Lipinski's Rule of Five. This rule is a set of guidelines for predicting the likelihood of a compound to become an orally active drug based on its absorption, distribution, metabolism, and excretion (ADME) properties. Drug likeness of the best docked phytochemicals, physical and structural properties MW, h-bond, oral bioavailability and solubility were predicted computationally with FafDrug4 [25].

## Molecular Dynamics Simulation:

To investigate stability under dynamic conditions, we performed a 100 ns molecular dynamics simulation for the PPARγ protein and the two previously screened PPARγ-phytocompound complexes. We compared these simulations with the dynamic behavior of the PPARγ complex to the full agonist rosiglitazone (PDB ID: 7AWC). Newtonian molecular dynamics simulations of the protein systems and other molecular systems generated trajectories of atom coordinates, velocities, and energies. Statistical analysis was carried out on these trajectories to obtain information about the systems. The simulations provided insights into the stability and structural changes of the ligands, proteins, and protein-ligand complexes through multiple trajectories. This procedure was performed using GROMACS 2018.2 software [26].

First, we began with the resulting structures from docking analysis. A complete GROMACS simulation typically involves six steps: topology generation, building a box, solvation, system energy minimization, system equilibration, and MD production. Next, trajectories were generated, and results were analyzed. Protonation and minimization steps were applied to the systems using the GROMOS96 43A1 force field. The molecular topology file parameters for the different ligands were generated using the PRODRG server [27]. The docked complexes were solvated using the SPC216 water model and immersed in water cubic boxes with a 12-Å margin distance. The system was neutralized, and the energy minimization was performed through 50,000 steepest descent steps with a maximum step size of 0.01 nm, maintaining a tolerance of 1000 kJ/mol/nm. Each system was then subjected to equilibration at 300 K and 1 bar for 100 ps under position restraints for heavy atoms and LINCS constraints for all bonds. Finally, the full system was subjected to a 100 ns MD simulation run, and the corresponding atom coordinates were stored every 0.002 ps during the simulation for later analyses. The resulting trajectories of simulated systems were saved for detailed analysis. The root-mean-square deviation (RMSD), root-mean-square fluctuation (RMSF), radius of gyration (Rg), solvent-accessible surface area (SASA), and the number of hydrogen bonds (H-bonds) were analyzed throughout the trajectory using the "gmx rms," "gmx rmsf," "gyrate," "gmx sasa," and "gmx hbond" built-in functions of the GROMACS software, respectively. Graph plotting was performed using GRACE software [28].

## Free binding energy calculation:

The MD studies were conducted for a period of 30 nanoseconds, and the binding free energy of the various complexes was subsequently calculated using the "g_mmpbsa" package. This software tool was developed using two popular open-source programs, GROMACS and APBS [29]. The MM-PBSA method was used to calculate the components of the binding energy, with the exception of the entropic term, and the energetic contribution of each residue to the binding was determined using an energy decomposition scheme. The resulting output was then used as an input for Python scripts to obtain the final binding energy.

## Results:

**Figure 1:**
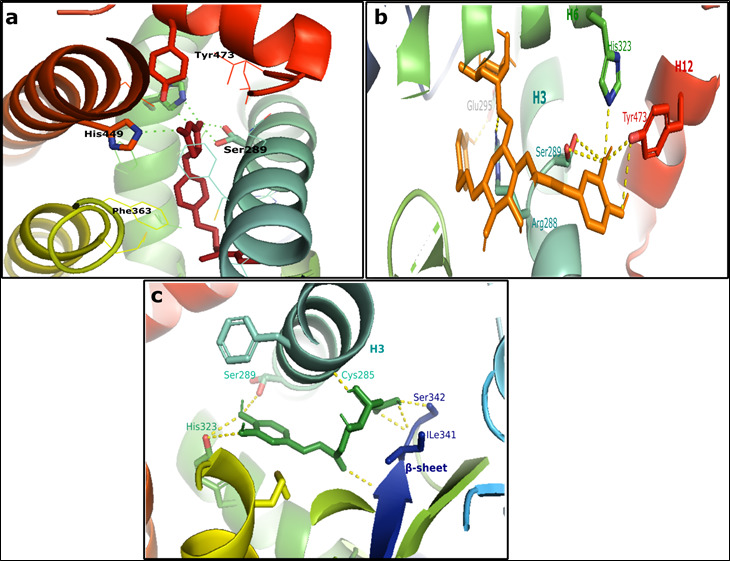
3D interaction of rosiglitazone and identified phytochemicals in the active site of PPARγ, H-bond shown as green and yellow dashes, (a) rosiglitazone in red; (b) 3,4-DICQA in orange color (c) Chlorogenic acid in green color .

**Figure 2:**
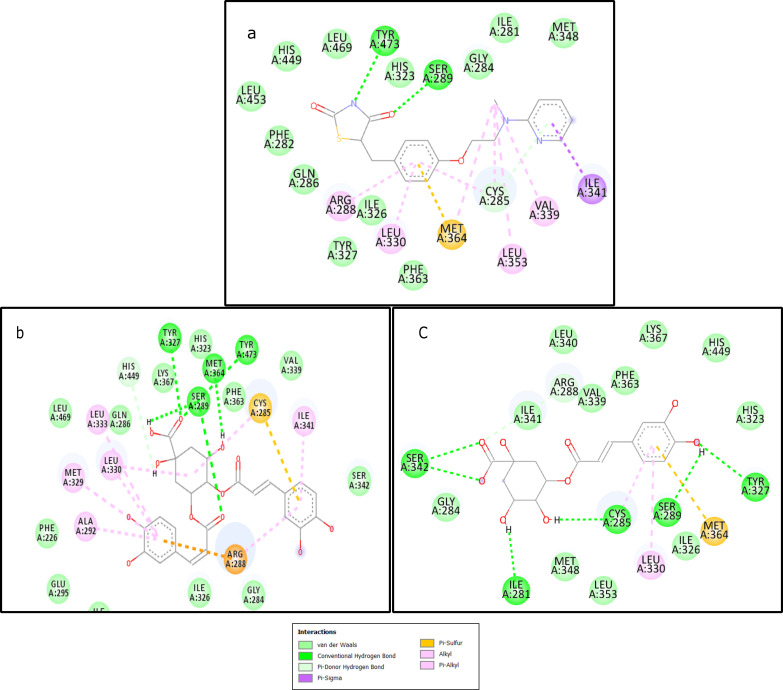
2D structures of the reference Roziglitazone and the two best docked phytochemicals (a) Rosiglitazone (b) 3,4-dicaffeoylquinic acid (c) Chlorogenic acid.

Database Screening and Molecular Docking The generated phytochemical ligands library was docked against PPARγ and the docked compounds were ranked based on a stringent filter that included factors such as the strength of hydrogen bonding and a robust network of hydrophobic bonds. Out of 600 docked phytochemicals, the top-ranking docking poses were selected. After analyzing the binding energy, two phytochemicals 3,4-dicaffeoylquinic acid (3,4-DICQA) and chlorogenic acid (CGA) were found to bind with strong affinity within the active site of the receptor and form a robust network of hydrogen and hydrophobic interactions (Figure 1, Table 2). In particular, 3,4-DICQA had a binding score of -8.2 Kcal/mol (Table 1 - see PDF) and was found to bind in the same pocket in the catalytic site as the full agonist rosiglitazone. It formed hydrogen bonds with a set of residues in the AF1 region, including Tyr473 and His449, as well as with Ser289, Tyr327, and Met364 of the ligand binding domain (Figure 1b). Additionally, Cys285 and Arg288 formed sulfur interactions with the benzene cycles of 3,4-DICQA, implicating strong hydrophobic interactions with residues in the ligand binding domain, including Ile341, Glu295, His323, Ser342, and Ile326 (Figure 2b). Chlorogenic acid was observed to bind through hydrogen bonds with the residues of the beta sheet, including Ser342 and Ile341, as well as with Ser289, Tyr327, Ile281, and Cys285, forming a network of hydrogen bonds within the active site. Strong hydrophobic and van der Waals interactions were also observed with other residues of the AF1 region of the ligand binding domain, such as His323 and His449, as illustrated in Figure 2.

## Drug-like properties of the identified hits:

3,4-DICQA and chlorogenic acid are two compounds of interest for drug discovery. When evaluated based on Lipinski's Rule of Five, 3,4-DICQA violates three rules: molecular weight and hydrogen bond donors, which may reduce its likelihood of being a successful orally active drug. In contrast, chlorogenic acids violate only one rule, hydrogen bond donors. Although this suggests a higher potential for these two compounds to be successful orally active drugs, it's important to remember that Lipinski's Rule of Five is a guideline with exceptions. Some compounds may still be successful drugs even if they do not strictly adhere to the rules, while others that meet the criteria may not be. The Rule of Five serves as a valuable tool in guiding the early stages of drug discovery, but it should not be considered an absolute determinant of success (Table 3).

## Dynamic molecular simulation:

**Figure 3:**
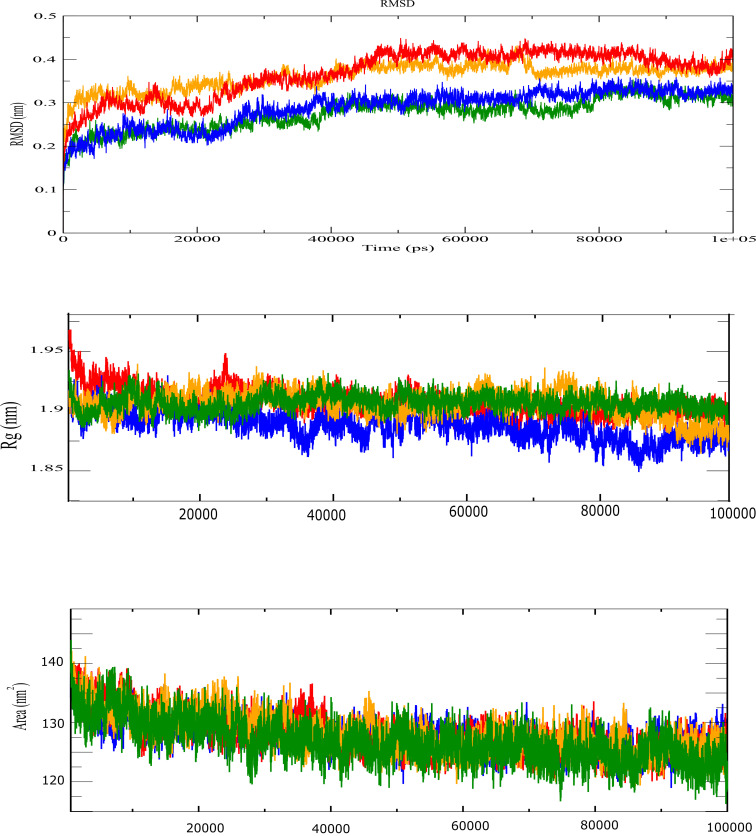
(a) RMSD plot of PPARγ complexes, (b) Radius of gyration plot of different PPARγ complexes with color-coded panels, and (c) Solvent-accessible surface area (SASA) plot of different PPARγ complexes with color-coded panels.The receptor binds to rosiglitazone is represented in red, 3,4-DICQA in orange color, and chlorogenic acid in green color.

**Figure 4:**
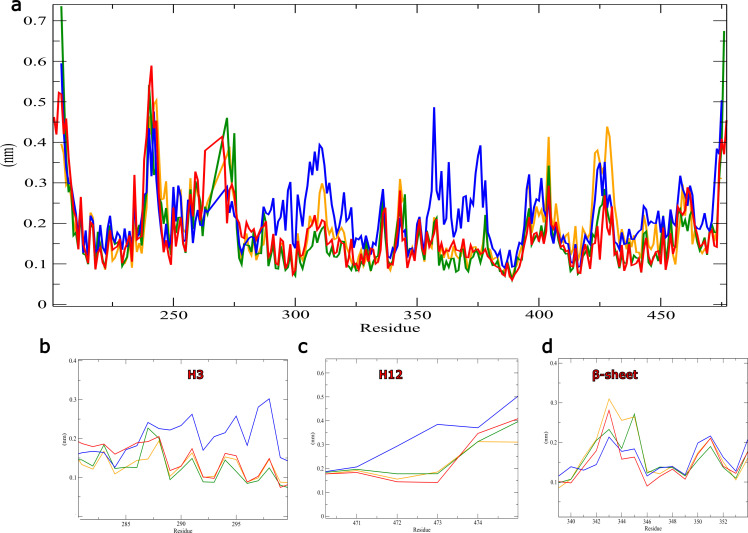
Root-mean-square fluctuations (RMSF) plot of PPARγ complexed with different complexes; phytochemicals (a), rosiglitazone, Apo PPARγ as a reference for the 100 ns simulation. (b) Fluctuation of helix 3 of PPARγ, (c) Fluctuation of H12, and (d) Fluctuation of beta-sheet.The receptor binds to rosiglitazone is represented in red, 3,4-DICQA in orange color, and chlorogenic acid in green color.

**Figure 5:**
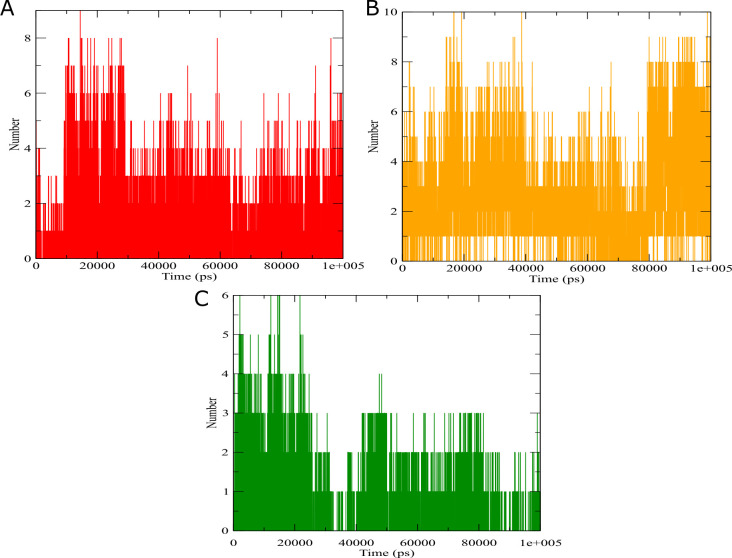
2D diagram of observed hydrogen bond patterns for the two different complexes and rosiglitazone as a reference of PPARγ during the 100 ns simulation.The receptor binds to rosiglitazone is represented in red, 3,4-DICQA in orange color, and chlorogenic acid in green color.

As demonstrated by the docking study, it showed the strongest affinity and a solid network of interaction with PPARγ. To assess the stability of the docking data and to investigate the validity of the results, the two Complexes (3,4-DICQA and Chlorogenic acid) were simulated with molecular dynamics for 100ns using GROMACS software, and for comparison reference structure of PPARγ-Rosiglitazone and PPARγ- alone without ligand was also subjected to 100 ns MD Simulation. Molecular dynamics was performed for 100 ns to analyze the stability and the H-bond of the five complexes. As a result we generated in Figure 3, Figure 4,Figure 5 the MD pathways for three complexes plus the PPARγ alone without ligand to reveal the changes occurring in the presence of identified modulators. The trajectories are superimposed on the top of each other, PPARγ-Rosiglitazone is represented in red color, PPARγ-3,4-DICQA in orange , PPARγ-Chlorogenic acid in green, PPARγ alone in blue color. For the PPARγ-Rosiglitazone and PPARγ-3,4-DICQA, the Cα-RMSD variation of both complexes varies between 0 and 0.48 nm (Figure 3a). Generally, no considerable variation of RMSD fluctuations between both complexes was observed, the RMSD variation was similar during 25ns-48ns and during 90ns to the last of the simulation. For the PPARγ-Chlorogenic acid and PPARγ alone , Both complexes displayed highly stability compared to full agonist rosiglitazone, the RMSD of both complexes varies around 0.22 and 0.38 ns during the 100 ns of the simulation (Figure 3a). Their RMSD variation was similar in both except for small fluctuations during 33ns-39ns and between 68ns-78ns where the RMSD variation of the PPARγ increased compared to PPARγ-Chlorogenic (Figure 3a). The global dimensions of the both PPARγ-3,4-DICQA and PPARγ-Chlorogenic acid showed the same variation as the complex of the rosiglitazone and all complexes remain stable around 1.9 nm during the simulation, except PPARγ alone which remain the small values of Rg around 1.86 nm (Figure 3b). The solvent-accessible surface area (SASA) was computed to evaluate the maintenance of protein packing in the system (Figure 3.c). The SASA analysis of all complexes showed a modest difference in area properties around 125-130 nm compared with the reference complex of rosiglitazone (between 127-130nm2), except PPARγ-Chlorogenic acid that showed low values around 120 nm (Figure 3c). The number of hydrogen bonds involved in all complex interactions varies between 0 and 10 during the100 ns of simulation (Figure 5). On the other hand (Figure 4) gives RMSF values of different complexes during 100 ns. Generally, the PPARγ alone residues showed high fluctuations compared to the full agonist and other complexes. Indeed, the Chlorogenic acid showed low values compared to other complexes (Figure 4.). In addition, the residues of the helix 12 showed less fluctuation values with rosiglitazone (0,15nm), with 3,4-DICQA and Chlorogenic acid (0,18), while the H12 in PPARγ alone show the high values of RMSF arrives to 0,54 nm suggesting that the interactions with 3,4-DICQA and Chlorogenic acid stabilize the H12 similar to the rosiglitazone (Figure 4.b). The observation of the flexibility value of beta sheet residues (340-351) showed that the receptor alone show the less values of RMSF (0,12-0,2nm) followed by Chlorogenic acid with values between 0,1nm and 0,27 nm, while beta sheet residues with the 3,4-DICQA and rosiglitazone (Figure 4.b) showed the high values of RMSF vary between (0,8-0,33nm) and (0,1-0,27 nm) respectively, thus speculates that the residues of beta sheet stabilized with the hydrogen bonds in Chlorogenic acid can achieve a stable interaction over the simulation time.

## Free binding energy:

A comparative analysis of the energetic parameters for phytochemicals interacting with peroxisome proliferator-activated receptor (PPAR gamma) showed that both chlorogenic acid (MNPDB00380) and 3,4-DICQA (MNPDB00479) exhibited stronger binding affinities compared to rosiglitazone. Chlorogenic acid had the strongest binding affinity with a binding energy of -139.442 kJ/mol, indicating the most robust interaction with the PPARγ while 3,4-DICQA followed closely with a binding energy of -124.179179 kJ/mol. Furthermore, chlorogenic acid (MNPDB00380) displayed the most favorable electrostatic energy (-58.928 kJ/mol), suggesting enhanced electrostatic interactions with the receptor, whereas 3,4-DICQA (MNPDB00479) demonstrated less favorable electrostatic energies relative to rosiglitazone. Regarding polar solvation energy, all compounds exhibited values in a similar range, with chlorogenic acid (MNPDB00380) presenting a slightly higher value (46.069 kJ/mol) than the others. In terms of van der Waals energy, chlorogenic acid (MNPDB00380) showed the most favorable value (-121.829 kJ/mol), implying strong nonpolar interactions with the receptor. Both rosiglitazone and 3,4-DICQA (MNPDB00479) displayed less favorable van der Waals energies. Analyzing SASA energy, chlorogenic acid (MNPDB00380) emerged as the most favorable candidate (-4.754 kJ/mol), possibly reflecting enhanced solvent exposure or conformational changes upon binding. Conversely, 3, 4-DICQA (MNPDB00479) displayed a less favorable SASA energy when compared to rosiglitazone. Overall, both chlorogenic acid and 3, 4-DICQA are recommended as potential candidates for further investigation as PPAR gamma activators.

## Discussion:

Over many centuries, natural products have been widely used as the major source of many disease treatments. Many plant-derived constituents and/ or extracts were used to treat complex diseases. Significant research efforts were and continue to be undertaken in order to explore the promising natural structures for drug discovery. As the incidence of metabolic disorders and particularly type 2 diabetes (T2D) keeps rising tremendously, the search for alternative and affordable medicines seems to alleviate the clinical burden of these diseases worldwide. Diabetes medicines target their multifactorial genesis to provide a therapeutic effect based on insulin secretagogues and insulin sensitizers [30]. Peroxisome proliferator-activated receptor gamma (PPARγ) is a nuclear receptor that plays a crucial role in the regulation of glucose and lipid metabolism, making it an essential component in the management of diabetes, metabolic syndrome, and cardiovascular diseases. Activation of PPARγ has been shown to enhance insulin sensitivity, promote glucose uptake in adipose tissue and skeletal muscle, and reduce inflammation, which are critical factors in the pathogenesis of type 2 diabetes and metabolic syndrome. Furthermore, PPARγ activation has been linked to improvements in endothelial function and a reduction in atherosclerosis, contributing to better cardiovascular health. The use of PPARγ agonists, such as thiazolidinediones, has proven effective in the treatment of these conditions by modulating PPARγ activity and targeting its downstream effects. Therefore, a deeper understanding of PPARγ and its activation is essential for the development of novel therapeutic strategies to combat diabetes, metabolic syndrome, and cardiovascular diseases [10], [30-31].

Computational methods have been proved through numerous works, as powerful methods in guiding the drug discovery of molecules capable of efficiently binding to biological targets, like proteins. These interactions can be exploited towards the discovery of protein modulators, or modulators in the human body. Our results are in line with this approach and suggest that the selected molecules have a high potential of binding affinity with PPARγ. Docking results showed that two molecules out of 600 phytochemicals had the top best docking scores (Table 1 - see PDF) and showed the strength of hydrogen bonding, and a robust network of hydrophobic bonds with functional residues, thus suggesting that they could activate the receptor. The structural mechanisms underlying the activation of PPARγ are well understood. Agonists stabilize an active state of the AF-2 surface (Tyr473, His449, and His323) by forming interaction with residues near helix 12 (residues 470–477). Full agonists of PPARγ form a direct hydrogen bond with Tyr473 on helix 12, consequently provoking transcriptional activation [31]. Partial agonists generally do not form a hydrogen bond with Tyr473, but they stabilize helix 12 through interactions with other regions of the ligand-binding pocket [31]. Additionally, a recent study indicates that the hydrogen bonding of the ligand to Arg288 could be a critical mediator of the selective PPARγ reverse agonism that seems promising for improving the therapeutic index associated with antidiabetic ligands of PPARγ [30].

The partial agonists are delimited by the H3, the β-sheet, and the ω loop (260-275). Here, Chlorogenic acid switches the binding structure of partial agonists within the identified Phytocompounds. The ligand occupy the ligand binding domain wich delimited principally by β-sheet and H3; in particular, it forms a combination of H-bonds with Arg288 of H3 and H-bonds with the backbone amide of Ser342, Glu343 and, as well as extensive van der Waals and hydrophobic interactions with Ile341 of the β-sheet and Ala292 of H3. This β sheet was not found in interaction with the full agonist (Figure 1), whereas we suggest that β sheet interaction could be responsible for compensating for the lack of H12 stabilization. In summary, this molecule CGA forms hydrogen bonds with Ser342, an amino acid related to partial agonist binding.

It also interacts with Ile341, Ser289, Tyr327, and Ile281. Based on its interaction with Ser342 and the absence of direct interactions with other full agonist-associated amino acids, Chlorogenic acid is likely a partial agonist. Chlorogenic acid (CGA), belongs to the hydroxycinnamic acid family and is formed by the esterification of caffeic acid and quinic acid [33] where it has been shown that CGA exerts hypoglycemic, hypolipidemic, antibacterial, antioxidant, and anti-inflammatory effects [33-34,35, 36]. A recent study showed that Chlorogenic acid and its derivatives can successfully ameliorate hyperglycemia and hyperinsulinemia and improve the function of the pancreas, and could be considered a promising medicine for diabetes treatment by restoring pancreatic function effectively [37]. In addition, CGA has been shown to modulate glucose and lipid metabolism in vivo, both in healthy individuals and in those with metabolic disorders [38]. The broad distribution and the remarkably pharmacological activities of these natural phenolic acids indicated their potential in the discovery and development of new natural drugs. CGA is produced in plants through the shikimic acid pathway during aerobic respiration. It can be found in the Moroccan medicine plant the Anabasis aretioides and Coriandrum sativum L [39],[40]. Furthermore, CGA has been identified as a potential PPARγ agonist, similar to roziglitazion, and has been shown to stimulate the expression of PPARγ, making it a potential insulin sensitizer and lipid-lowering agent [41]. A study conducted in 2017 suggested that CGA has suitable physicochemical properties to be considered a lead bioactive molecule for the development of novel agents with similar properties [42]. The study also revealed that CGA could bind to PPARγ and could activate its expression. 3,4-DICQA (MPDB ID: MNPDB00479): This molecule forms hydrogen bonds with Tyr473, Ser289, and His449, all of which are linked to full agonist binding. Moreover, it interacts with Met364 and Tyr327. Considering its interactions with amino acids typically associated with full agonists, 3,4-dicaffeoylquinic acid may also be a full agonist.The MD simulations confirmed the docking studies since all tested molecules tended to stay bound to PPARγ. 3,4-DICQA, Chlorogenic acid (CA) have shown the best results in MD simulations and MM-PBSA calculations, indicating, they have better stability and could be potent modulators against PPARγ. This study identified three high potential conductive phytochemicals capable of binding to the active site of PPARγ. The set of compounds identified can lead to a therapeutic solution against DT2 by effectively targeting the function site of PPARγ. Rosiglitazone is a full agonist of the peroxisome proliferator-activated receptor gamma (PPARγ), and although it is effective in treating metabolic disorders, it has been associated with a higher risk of adverse effects compared to partial agonists. Some of the adverse effects linked to rosiglitazone use include weight gain, fluid retention, bone fractures, and an increased risk of heart failure. Moreover, rosiglitazone has been found to increase the risk of cardiovascular events such as heart attacks and strokes, leading to regulatory restrictions on its use in some countries [32]. In contrast, partial agonists have a better safety profile and a lower risk of adverse effects. They have also been found to have a lower risk of cardiovascular events and are generally well-tolerated by patients. The results of MMPBSA (Table 3) indicate that complexes are bonded by the PPARγ protein efficiently. Therefore, the bioactivity of 3,4-DICQA, Chlorogenic acid are worth further experimental work for structure-based lead optimization. Our natural compounds could serve as promising candidates for the treatment development of T2D and establish a basis for designing specific drugs targeting PPARγ with properties over the current TZD drugs family. All the three complexes showed higher values in the relative fluctuation in the RMSD compared with the receptor complexed to rosiglitazone thus suggesting the stability of the PPARγ unbounded in physiological conditions. However, the higher RMSD observed in the five complexes may explain why the structural and conformation change when provided by the different ligands. However, all complexes showed relatively similar and consistent stability throughout the MD simulation of 100 ns. The minor variation in the value of the radius of gyration compared to the reference PPARγ suggests that the protein is compactly packed, and the binding of ligands does not affect the rigidity of the protein. The SASA and hydrogen bonding results support the stable binding of the small Phyto compound in the protein.

## Conclusion:

Moroccan plant extracts have been shown to exhibit antimicrobial, anticancer, and antidiabetic activity. However, the therapeutic properties of most of these compounds have not yet been fully studied as Indian and Chinese ones. In this context, we conducted literature research to extract Moroccans compounds with their 3D structures to reveal their molecular impact against PPARγ using advanced computer-aided drug discovery approaches. From the results of molecular docking and MD simulations with MM-PBSA calculations, we have concluded that 3,4-DICQA interacts similarly with full agonists and Chlorogenic acid binds as partial agonists based on structural analysis of dynamic molecular simulation compared to rosiglitazone dynamic and previous studies. The two compounds might improve insulin sensitivity through PPARγ. Hence, we suggest that two phytochemicals that may be used for PPARγ activation and may be further modified and synthesized to develop potential drug candidates against metabolic syndrome and especially T2DM.

## Data availability:

All data generated or analyzed during this study are included in this published article, and binding affinity results is available in the website www.mpdb.org

## Figures and Tables

**Table 2:** Results of top docked against PPARγ, with their respective binding affinity, hydrogen and hydrophobic interacting residues.

	**MW**	**log P**	**HBA**	**HBD**	**Solubility**	**Oral Bio availability**	**Result**
3,4-DICQA MNPDB00479	516.45	1.52	12	7	Good	Good	Accepted
Chlorogenic acid MNPDB00380	354.31	-0.42	9	6	Good	Good	Accepted

**Table 3:** Mean and standard deviation values of the complexes binding energies calculated by the g_mmpbsa methods

**Compound name**	**Binding energy**	**Electrostatic energy**	**Polar solvation energy**	**van der Waal energy**	**SASA energy**
Rosiglitazone	-120.329kJ/mol	-40.802 kJ/mol	36.747kJ/mol	-106.221kJ/mol	-10.053kJ/mol
3,4-DICQA MNPDB00479	-124.179179 kJ/mol	-36.747 kJ/mol	36.822 kJ/mol	-112.053kJ/mol	-12.201 kJ/mol
Chlorogenic acid MNPDB00380	-139.442 kJ/mol	-58.928 kJ/mol	46.069kJ/mol	-121.829kJ/mol	-4.754kJ/mol

## References

[1] Alberti K (1998). Diabet Med.

[2] https://pubmed.ncbi.nlm.nih.gov/11368702/.

[3] Einhorn D (2003). Endocr Pract Off J Am Coll Endocrinol Am Assoc Clin Endocrinol.

[4] Alberti K (2005). Lancet Lond Engl.

[5] Wilson P (2005). Circulation.

[6] Alberti K (2009). Circulation.

[7] Serafino-Agrusa L (2015). World J Clin Cases WJCC.

[8] Caterson I (2004). Circulation.

[9] Rochlani Y (2017). Ther Adv Cardiovasc Dis.

[10] Berger JP (2005). Trends Pharmacol Sci.

[11] Monsalve FA (2013). Mediators Inflamm.

[12] Stump M (2015). Curr Hypertens Rep.

[13] Mansour M (2014). Prog. Mol. Biol. Transl. Sci..

[14] Algandaby MM (2020). Saudi J Biol Sci.

[15] Fitzgerald ML (2002). J Mol Med Berl Ger.

[16] Lalloyer F, Staels B (2010). Arterioscler Thromb Vasc Biol.

[17] https://www.ncbi.nlm.nih.gov/books/NBK67752/.

[18] Martens FMAC (2002). Drugs.

[19] Koshiyama H (2000). Lipoprotein Metabolism and Atherogenesis.

[20] Werner AL, Travaglini MT (2001). Pharmacotherapy.

[21] Phillips LS (2001). Diabetes Care.

[22] Kaul S, Diamond GA (2008). Curr Atheroscler Rep.

[23] Trott O, Olson AJ (2010). J Comput Chem.

[24] https://discover.3ds.com/discovery-studio-visualizer-download.

[25] Lagorce D (2017). Bioinforma Oxf Engl..

[26] Pronk S (2013). Bioinformatics.

[27] Schüttelkopf AW, van Aalten DMF (2004). Acta Crystallogr D Biol Crystallogr.

[28] https://plasma-gate.weizmann.ac.il/Grace/.

[29] Genheden S, Ryde U (2015). Expert Opin Drug Discov..

[30] Brust R (2018). Nat Commun.

[31] Berger J, Moller DE (2002). Annu Rev Med.

[32] Nissen SE, Wolski K (2007). N Engl J Med..

[33] Wang L (2022). Front Nutr..

[34] Yan Y (2020). J Immunol Res..

[35] Li SY (2009). Biomed Environ Sci BES..

[36] Lou Z (2011). J Food Sci..

[37] Wu L (2007). J Zhejiang Univ Sci B..

[38] Meng S (2013). Evid-Based Complement Altern Med ECAM..

[39] Berrani A (2018). J Basic Clin Physiol Pharmacol..

[40] Msaada K (2017). Arab J Chem..

[41] Peng SG (2018). BioMed Res Int..

[42] Sanchez MB (2017). Biomed Pharmacother Biomedecine Pharmacother..

